# Supplementary material for: A novel fungal gene regulation system based on inducible VPR-dCas9 and nucleosome map-guided sgRNA positioning
Source: Appl Microbiol Biotechnol. 2020 Oct 2;104(22):9801–22. doi: 10.1007/s00253-020-10900-9 (PMC7595996; doi:10.1007/s00253-020-10900-9)
Supplement: Supplementary file 1 — (PDF 1562 kb) [file 253_2020_10900_MOESM1_ESM.pdf]

Applied Microbiology and Biotechnology

A novel fungal gene regulation system based on inducible VPR-dCas9 and nucleosome map-guided sgRNA positioning.

Supplemental Materials

Andreas Schüller[1], Lisa Wolansky[1], Harald Berger[1], Lena Studt[1], Agnieszka Gacek-Matthews[1§], Michael Sulyok[2], and Joseph Strauss[1*]

[1]Institute of Microbial Genetics, Department of Applied Genetics and Cell Biology, [2] Institute of Bioanalytics and Agrometabolomics, Department of Agrobiotechnology, BOKU-University of Natural Resources and Life Sciences Vienna, BOKU-Campus Tulln, Tulln/Donau, Austria.

[§]present address: Institute of Microbiology, Functional Microbiology Division, University of Veterinary Sciences Vienna.

*Corresponding Author: Joseph.strauss@boku.ac.at

Address: Joseph Strauss, Fungal Genetics Lab, Institute of Microbial Genetics, Department of Applied Genetics and Cell Biology, BOKU-University of Natural Resources and Life Sciences Vienna, BOKU-Campus Tulln, Konrad Lorenz Strasse 24, A-3430 Tulln/Donau, Austria. Tel. +43-147654-94420

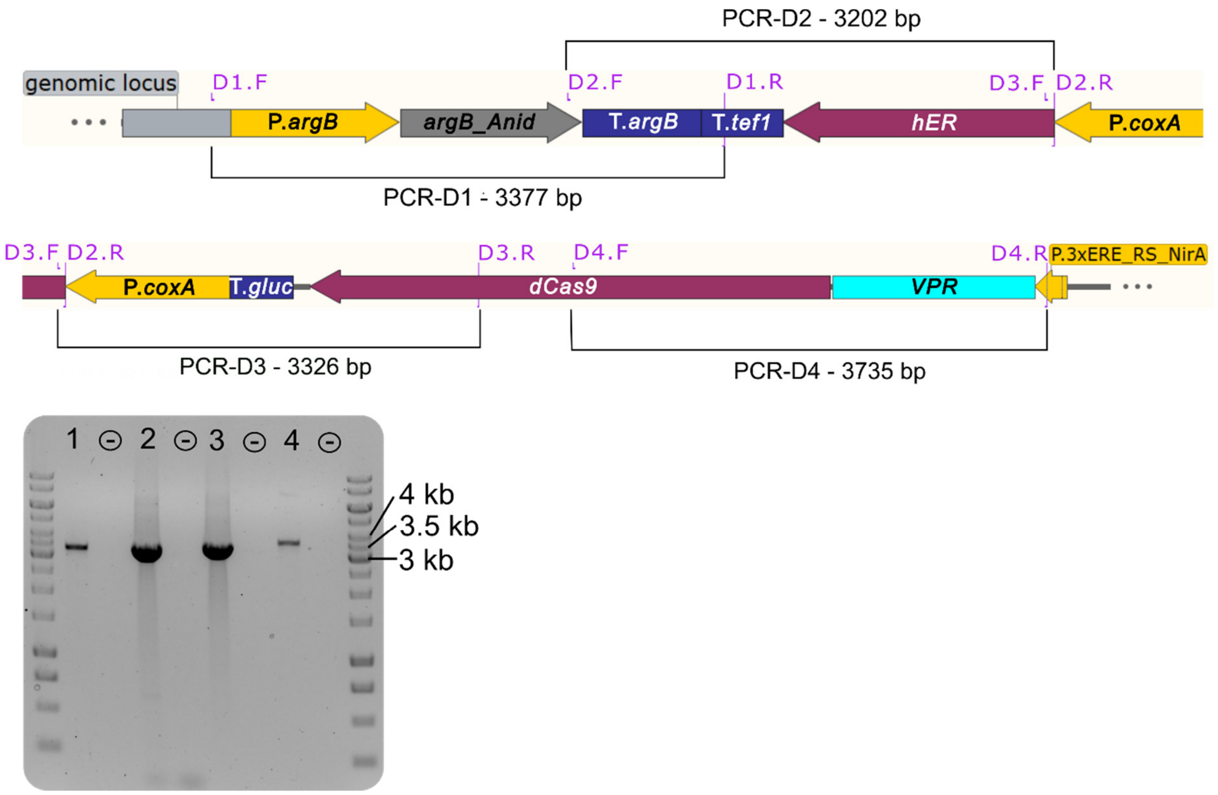

Fig.S1 <u>Diagnostic PCRs of Strain VPR4.</u> The 5’ part of plasmid VPR4 is depicted as it would integrate into the desired genomic locus (*argB*). The “genomic locus” at the 5’ of the construct (in grey) is the upstream region of the *argB* gene that is not present on the plasmid VPR4. Primer pairs (violet) (Dx.F and Dx.R (x=1-4)) are connected by a black lines which correspond to the PCR product (D1- D4). The results of the diagnostic PCR for Strain VPR4 can be retrieved from the gel picture where each amplicon (1= D1, 2= D2, etc.) is followed by a negative control (-). Hence, Strain VPR4 has plasmid VPR4 in the desired locus (*argB*) and possesses all necessary features for the expression of the activator VPR-dCas9. See Supplementary Table S1 and Fig.1 for more detailed information about primers and the plasmid respectively

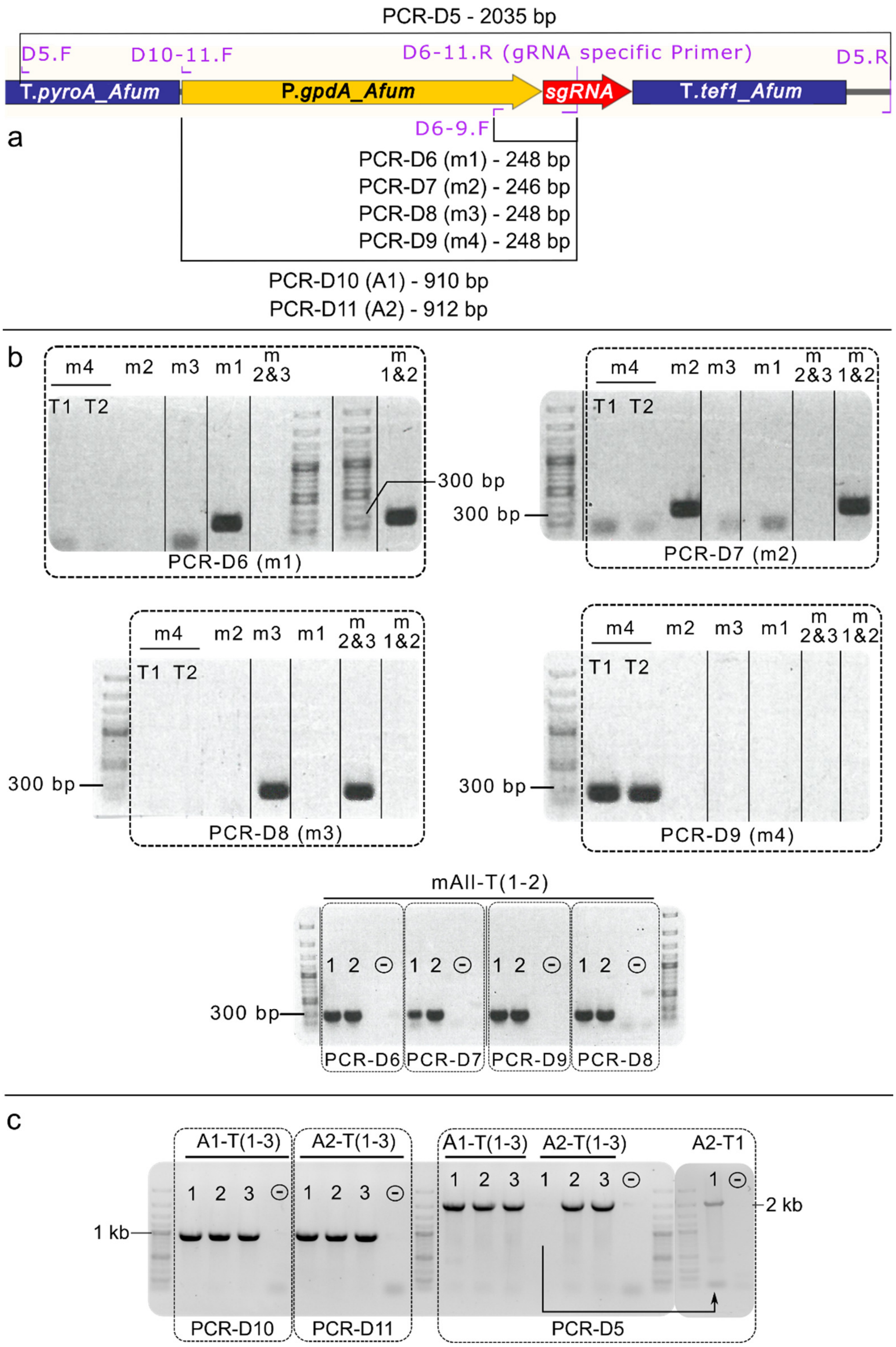

Fig.S2 Diagnostic PCRs of single guide RNA carrying strains. **a.** Section of the plasmid map containing the sgRNA cassette. Primer pairs (violet) (Dx.F and Dx.R (x=5-11)) are connected by a black line which correspond to the PCR product (D5- D11). The results of the diagnostic PCR for Strain VPR4 can be retrieved from the gel pictures in **b** (activation strains for *mdpE*) and **c** (activation strains for AN8506 and AN8507). The strain description was abbreviated by omitting "VPR4-" (i.e. A1-T1 corresponds to VPR4-A1-T1). The abbreviated strain names are displayed above the respective lanes. Each box (dashed lines) represents the results of a primer pair (bottom side of the box). Solid lines separating lanes indicate that dispensable lanes of the gel picture were removed. Information about the primer pairs are included in Supplementary Table S1

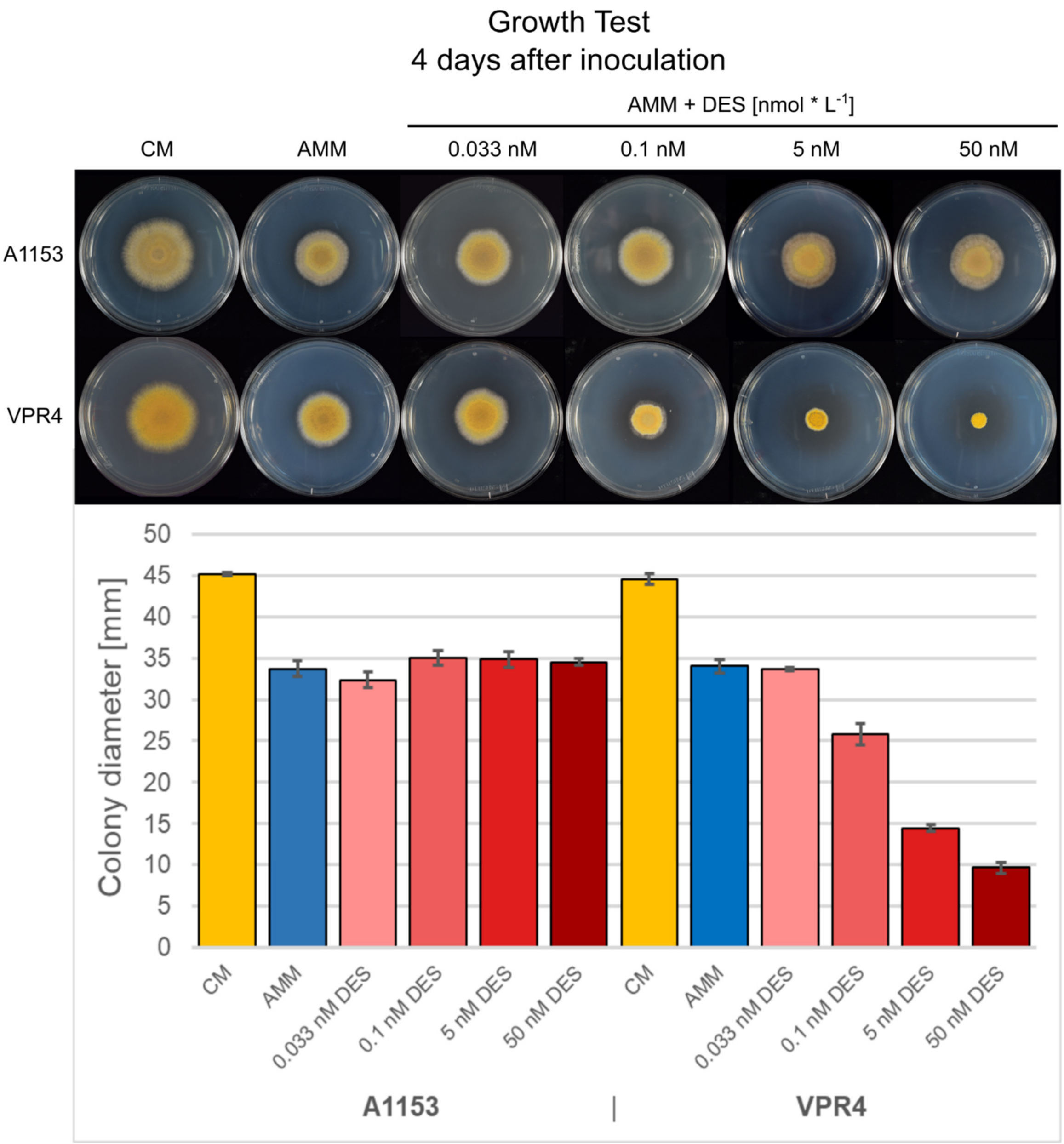

Fig.S3 Growth Test. Impact of inducer DES on the recipient strain FGSC A1153 and strain VPR4. Only strains that carry the *hER* are affected by the inducer DES. The recipient strain does not show any phenotypical changes upon incubation with DES. Activation of the human estrogen receptor (Strain VPR4 and descendants) leads to growth phenotypes. At 33 pM DES (concentration for activation experiments), no impact on radial growth or morphology can be observed. At 100 pM DES, a significant radial growth reduction of ~ 40% can be observed. At 50 nM DES, the fungus does not pass the inoculation radius but still can sporulate (radial growth reduction of 100%). Inoculations were done in triplicate and growth was measured after a time period of 4 days

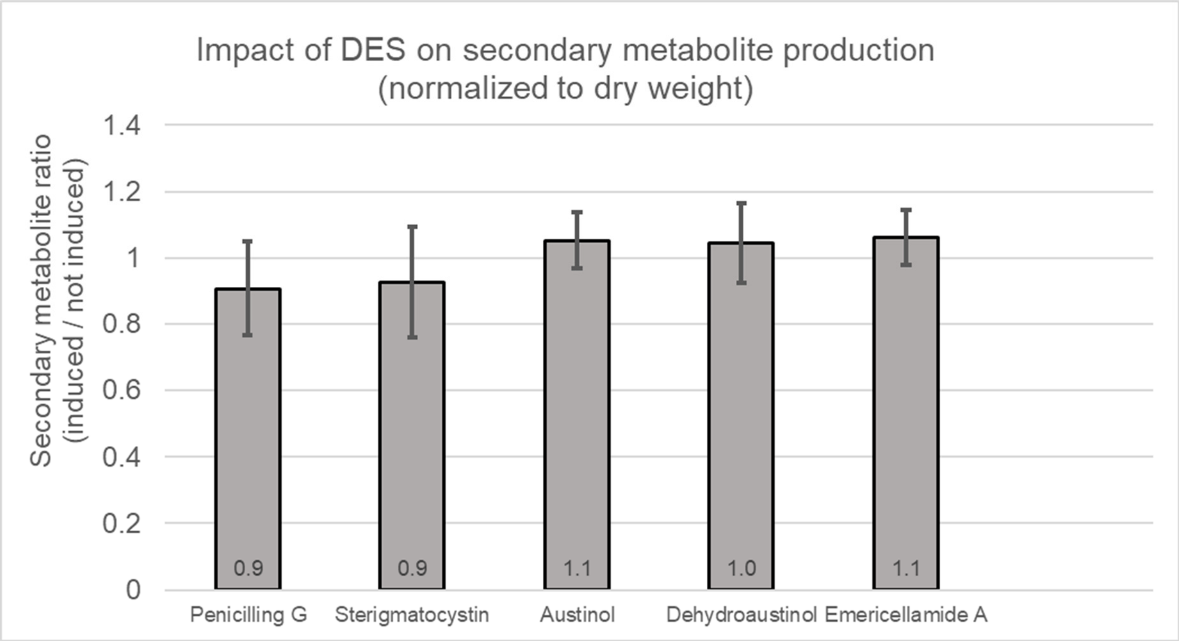

Fig.S4 Impact of DES addition on the production of SMs in a hER carrying strain. Supernatants for chemical analysis of *A. nidulans* SMs were taken 8 hours after induction (i.e. 48 hours total inoculation time). SM production was normalized to dry weight accumulation which was not influenced by DES addition. SM production was calculated by dividing the SM levels in the DES-induced by the non-induced state. All strains carry the *hER* gene and the fusion gene *VPR-dCas9*. The dataset comprises 3 different experiments with a total sample count of 22 (i.e. 11 times induced and 11 times not induced) The SM profile shows no significant change.

Table S1 Primer sets used during this study

Fragment	Primer sequences (5'-->3'): forward + reverse	Info / Purpose
	Primer pairs for yeast recombinational cloning	
argB	ggtaacgccagggttttcccagtcacgacgtctagagtctgctctcccagg	*argB_Anid*
	atgccattttcaccctgcaccagctctagactgatgatgcagtggttcg	
T.tef1-1	gctggtgcagggtgaaaatgg	T.*TEF1_Anig*
	cagagggtttccctgccacagtctgagttgggctacatacttggatggc	
hER	tcagactgtggcagggaaaccc	*hER*
	atgaccatgaccctccacacc	
P.*coxA*-1	gctttggtgtggagggtcatggtcattgtcctggtgggtgggttgc	P.*coxA_Anig*
	ccatattggttcaaatgtcgggtcagcgatagggctggtggtgcagg	
P.*coxA*-2	cctgcaccaccagccctatcgctgacccgacatttgaaccaatatgg	P.*coxA_Anig*
	accgaagaggatgggaaccc	
T.*gluc*	gggttcccatcctcttcggtatcttgttggggggaaggggttgtcaaatc	T.*gluc_Bcin*
	cgtatgtagataagatgtatg	
dCas9-1	tcatacatcttatctacatacgggtaaccctagacctttctcttcttctttggatgc<br>gatcg	*dCas9*
	Ggaacttcgaggaagtcgtggataaggg	
dCas9-2	ccttatccacgacttcctcgaagttcc	*dCas9*
	ggtagcggcagcggtagcggaatggacaagaagtactccattgg	
VPR	ttccgctaccgctgccgctaccaaacagagatgtgtcgaagatgg	*VPR*
	tctgtctgggcttgatttaccatggaggccagcggttccggacg	
P.ERE	cccgtccggaaccgctggcctccatggtaaatcaagcccagacagatatgttcg	p.ERE-RS-*nirA*
	gataacaatttcacacaggaaacagcggatcccccgggctgcaggaattc	
2µ-ori	ttcaaggagcgaaaggtgg	*2µ ori* 1/2
	ctgtacggtatacacatctaagctgcatgtgtcagaggttttca	
pyroA	tgaaaacctctgacacatgcagcttagatgtgtataccgtacag	*pyroA_Afum*
	tgtccagttgcatcgcactaagctcgagtcattacgcggagatgtgaagg	
P.*gpdA*	cttagtgcgatgcaactggac	p.*gpdA_Afum*
	tgtgtagattcgtctggtactg	
sgRNAscaff	gtaccagacgaatctacacaggtaaccgttttagagctagaaatagc	sgRNA-Scaffold
	aaatttttcactctccagccctgtcccattcgccatgccgaagc	
T.*tef1-2*	agggctggagagtgaaaaattttgc	T.*tef1_Afum*
	gcttacagacaagctgtgaccgtccattgttatcttcaatgtc	
URA3	gacattgaagataacaatggacggtcacagcttgtctgtaagc	*URA3*
	ttagttttgctggccgcatc	
	Primer pairs for transformant verification	
PCR-D1	ctacgtcgtgtacgttgag	in loco verification
	gctacaaaatgcctacgatgc	
PCR-D2	caaccggtcacttgtcttcc	*hER*
	atgaccatgaccctccacacc	

PCR-D3	cgttcccttggatctgatgc	*dCas9* 1/2
	cagatcaccaagcacgtgg	
PCR-D4	ttccactctgcttgtctcgg	*dCas9* 2/2 + *VPR*
	ggaaacacgccgagcgtc	
PCR-D5	ttcgacgcgaacgacatcg	sgRNA Cassette
	tgccgcatagttaagccagc	
PCR-D6	ctcctctcttcccatcatcagc	sgRNA m1
	acttggagtctctaaactccg	
PCR-D7	ctcctctcttcccatcatcagc	sgRNA m2
	gcaatgctcatgatccttg	
PCR-D8	ctcctctcttcccatcatcagc	sgRNA m3
	ttcgaccgataactccatctg	
PCR-D9	ctcctctcttcccatcatcagc	sgRNA m4
	ccttcatcaacgggaaacc	
PCR-D10	cttagtgcgatgcaactggac	sgRNA A1
	accctctggtagtcctctcc	
PCR-D11	cttagtgcgatgcaactggac	sgRNA A2
	gctagataccccggtgtttt	
Primer pairs for RT-qPCR		
actA-control	GAGGAAGTTGCTGCTCTCG	Check for residual gDNA
	GCTTGGACTGTGCCTCATCACCG	
actA-qPCR	CACCGGTATCGTCCTTGACT	-
	CTCAGCGGTAGTGGAGAAGG	
benA	CATGGCTGCCTCTGACTTCCG	-
	GCGCATCTGGTCCTCAACCTC	
dCas9	CAGGGATAAGCCCATCAGGGAGC	-
	TCAGTGTGGCGTCCAGGACC	
mdpE	CGAACTGCATCGCGTTCAACG	-
	ACAATCAACCCCTCCGCAATCG	
mdpG	GCACCGGCGTCAGTTACTCCAG	-
	CCAAAGCCCAGCGCAGC	
AN8506	CGGTCCCTACTACAATGCACG	-
	CTGCGGCATATCCAAGTCCAG	
AN8507	CGGCGAGAAGTAGTGGATCG	-
	CCTCCTTTCCGACGCTTTCG	
AN8504	CGCAATGCACCCCCTAACG	-
	CTGCTCTGCCCGACTGTGAC	

Table S2 Oligonucleotides for cloning of sgRNA carrying plasmids (descendants of *p*sgRNA). Forward and reverse oligonucleotide consist of overhangs (black, underlined letters; needed for YRC), inverted repeat of 5' protospacer (in red, bold letters needed for proper Hammerhead ribozyme function), Hammerhead motif (blue letters) and the protospacer sequence (red letters; i.e. target locus specific nucleotide sequence). Prior to YRC, the oligonucleotides had to be annealed as described in section "Plasmid generation".

Plasmid	Oligonucleotide sequence (5'-->3') (Forward + Reverse)
*p*sgRNA-m1	gtaccagacgaatctacaca**AACTCC**ctgatgagtccgtgaggacgaaacgagtaagctcgtcGGAGTTTAGAGACTCCAAgttttagagctagaaatagc
	gctatttctagctctaaaacTTGGAGTCTCTAAACTCCgacgagcttactcgtttcgtcctcacggactcatcag**GGAGTT**tgtgtagattcgtctggtac
*p*sgRNA-m2	gtaccagacgaatctacaca**ATCCTT**ctgatgagtccgtgaggacgaaacgagtaagctcgtcAAGGATCATGAGCATTGCCgttttagagctagaaatagc
	gctatttctagctctaaaacGGCAATGCTCATGATCCTTgacgagcttactcgtttcgtcctcacggactcatcag**AAGGAT**tgtgtagattcgtctggtac
*p*sgRNA-m3	gtaccagacgaatctacaca**CCATCT**ctgatgagtccgtgaggacgaaacgagtaagctcgtcAGATGGAGTTATCGGTCGAAgttttagagctagaaatagc
	gctatttctagctctaaaacTTCGACCGATAACTCCATCTgacgagcttactcgtttcgtcctcacggactcatcag**AGATGG**tgtgtagattcgtctggtac
*p*sgRNA-m4	gtaccagacgaatctacaca**AAACCG**ctgatgagtccgtgaggacgaaacgagtaagctcgtcCGGTTTCCCGTTGATGAAGgttttagagctagaaatagc
	gctatttctagctctaaaacCTTCATCAACGGGAAACCGgacgagcttactcgtttcgtcctcacggactcatcag**CGGTTT**tgtgtagattcgtctggtac
*p*sgRNA-A1	gtaccagacgaatctacaca**CTCCAT**ctgatgagtccgtgaggacgaaacgagtaagctcgtcATGGAGAGGACTACCAGAGGgttttagagctagaaatagc
	gctatttctagctctaaaacCCTCTGGTAGTCCTCTCCATgacgagcttactcgtttcgtcctcacggactcatcag**ATGGAG**tgtgtagattcgtctggtac
*p*sgRNA-A2	gtaccagacgaatctacaca**GCCCTA**ctgatgagtccgtgaggacgaaacgagtaagctcgtcTAGGGCTAGATACCCCGGTgttttagagctagaaatagc
	gctatttctagctctaaaacACCGGGGTATCTAGCCCTAgacgagcttactcgtttcgtcctcacggactcatcag**TAGGGC**tgtgtagattcgtctggtac
